# Modelo para mejorar la anemia y el cuidado infantil en un ámbito rural del Perú

**DOI:** 10.26633/RPSP.2017.112

**Published:** 2017-11-17

**Authors:** Juan Mansilla, Alvaro Whittembury, Robert Chuquimbalqui, Miriam Laguna, Vladimir Guerra, Ysela Agüero, Julia Piscoya, Jorge O. Alarcón

**Affiliations:** 1 World Vision Perú World Vision Perú Lima Perú World Vision Perú, Lima, Perú.; 2 Universidad Nacional Mayor de San Marcos Universidad Nacional Mayor de San Marcos Lima Perú Universidad Nacional Mayor de San Marcos, Lima, Perú.

**Keywords:** Child care, infant care, growth and development, anemia, Peru, Cuidado del niño, cuidado del lactante, crecimiento y desarrollo, anemia, Perú, Cuidado da criança, cuidado do lactente, crescimento e desenvolvimento, anemia, Peru

## Abstract

**Objetivo.:**

*Determinar la efectividad de la Estrategia para el Crecimiento y Desarrollo Integral (ECDI) de World Vision Perú sobre la anemia, desnutrición, desarrollo psicomotor y cuidado y protección infantil en niños menores de tres años de edad que residían en tres comunidades rurales de la Región Ayacucho del Perú*.

**Métodos.:**

*Se realizó un estudio cuasiexperimental en tres distritos rurales de la región Ayacucho, Perú. La medición de anemia se realizó con sangre capilar y el analizador HemoCue®, con ajuste de los valores de hemoglobina según a la altitud. La desnutrición se midió a través de los indicadores antropométricos de talla y peso; para ello se utilizó la herramienta Anthro de la Organización Mundial de la Salud. El desarrollo psicomotor se midió mediante el Test Abreviado del Ministerio de Salud del Perú. El cuidado y protección de los niños se midió a través de una ficha especialmente elaborada para este fin. Se realizaron tres mediciones, la línea basal en mayo del 2013, la medición intermedia en noviembre del mismo año y la final en mayo del 2014. En total, 283 niños tuvieron dos o más mediciones incluida la medición final y 205 tuvieron las tres mediciones*.

**Resultados.:**

*El análisis multivariado para medidas repetidas mostró una efectividad estimada de la ECDI para reducir la anemia de 33,1% (intervalo de confianza del 95%: 1,0%-54,7%) ajustada para la edad, sexo, consumo de alimentos ricos en hierro, consumo de alimentos potenciadores de la absorción de hierro, consumo de alimento inhibidores de la absorción de hierro, haber recibido suplementación de hierro en los últimos seis meses y haber participado del Programa Cuna Más*.

**Conclusiones.:**

*La ECDI fue efectiva para mejorar la nutrición de los menores de 36 meses de edad a través de la reducción de la anemia y el incremento del consumo de potenciadores de la absorción de hierro. Las intervenciones que incluyen componentes educativos y de seguimiento comunitarios podrían ser de gran ayuda para combatir la anemia en los niños menores de 36 meses de edad en comunidades rurales*.

## Abstract (en)

**Objective.:**

Determine the effectiveness of the Integrated Growth and Development Strategy (ECDI, Spanish acronym) of World Vision Peru on anemia, malnutrition, psychomotor development, and child care and protection in children aged <3 years living in three rural communities of Ayacucho Region, Peru.

**Methods.:**

A quasi-experiment was carried out in three rural districts of Ayacucho Region, Peru. Anemia was measured using capillary blood and the HemoCue® analyzer, with hemoglobin values adjusted for altitude. Malnutrition was measured with anthropometric indicators for height and weight, using the World Health Organization’s Anthro software. Psychomotor development was measured with the Abbreviated Test of the Ministry of Health of Peru. Child care and protection was measured using a form specifically designed for this purpose. Three measurements were carried out: baseline in May 2013, midpoint in November 2013, and final in May 2014. In total, 283 children had two or more measurements including the final measurement, and 205 had all three measurements.

**Results.:**

Multivariate analysis for repeated measures showed that ECDI had an estimated 33.1% effectiveness in reducing anemia (95% CI: 1.0%-54.7%), adjusted for age, sex, consumption of iron-rich foods, consumption of iron-absorption-enhancing foods, consumption of iron-absorption-inhibiting foods, having received iron supplementation in the last six months, and having participated in the *Cuna Más* Program.

**Conclusions.:**

ECDI was effective in improving nutrition in children aged <36 months through anemia reduction and increased consumption of iron-absorption enhancers. Interventions that include educational and community monitoring components could be of great assistance to combating anemia in children aged <36 months in rural communities.

## RESUMO

**Objetivo.:**

Determinar a efetividade da Estratégia de Crescimento e Desenvolvimento Integral (ECDI) da World Vision Peru para anemia, desnutrição, desenvolvimento psicomotor e atenção e proteção infantis em crianças menores de 3 anos de idade em três comunidades rurais.

**Métodos.:**

Foi realizado estudo quase-experimental em três distritos rurais da região de Ayacucho, no Peru. A avaliação da anemia foi realizada em sangue capilar com o uso do analisador HemoCue®, com correção dos valores de hemoglobina segundo a altitude. A desnutrição foi avaliada segundo indicadores antropométricos de tamanho e peso, com o uso da ferramenta Anthro elaborada pela Organização Mundial da Saúde (OMS). O desenvolvimento psicomotor foi avaliado com o Teste Abreviado do Ministério da Saúde do Peru. A atenção e a proteção infantis foram avaliadas com o uso de uma ficha elaborada para esta finalidade. Foram realizadas três avaliações, com a linha de base em maio de 2013, a avaliação intermediária sendo realizada em novembro deste mesmo ano e a avaliação final, em maio de 2014. Ao todo, 283 crianças passaram por duas ou mais avaliações, incluindo a avaliação final, e 205 passaram por três avaliações.

**Resultados.:**

Na análise multivariada de medidas repetidas, a efetividade estimada da ECDI de reduzir a anemia foi de 33,1% (intervalo de confiança de 95%: 1,0%-54,7%), corrigida para idade, sexo, consumo de alimentos ricos em ferro, consumo de alimentos potenciadores da absorção de ferro, consumo de alimentos inibidores da absorção de ferro, suplementação de ferro nos últimos seis meses e participação no Programa Cuna Más (programa nacional de desenvolvimento infantil).

**Conclusões.:**

A ECDI demonstrou efetividade para melhorar a nutrição das crianças menores de 36 meses com a redução da anemia e o aumento do consumo de potenciadores da absorção de ferro. Intervenções com componentes educacionais e acompanhamento comunitário podem contribuir consideravelmente para combater a anemia em crianças menores de 36 meses em comunidades rurais.

La infancia es un período muy importante para el desarrollo del individuo. En el Perú, las intervenciones públicas y privadas aún no disponen de estrategias integradoras que permitan atender a través de su oferta la promoción del desarrollo y el crecimiento infantil(1).

Las Encuestas Demográficas y de Salud Familiar (ENDES) del Perú reportan altas tasas de prevalencia de problemas nutricionales como la desnutrición crónica infantil (DCI) y la anemia en menores de cinco años. Si bien entre 2007 y 2012 la DCI se redujo de 28,5% a 18,6% (2, 3) aún existen grandes diferencias por departamentos, ya que aquellos con un alto porcentaje de población rural, como Huancavelica y Ayacucho, presentan DCI en el 54,2% y 35,3%, respectivamente. Esto contrasta con los departamentos de la costa, que tienen un bajo porcentaje de población rural y que se encuentran por debajo del promedio nacional de DCI.

Para este mismo período, el porcentaje de niños de 6 a 35 meses de edad con anemia disminuyó de 56,8% a 44,5%, aunque la zona rural fue también la de mayor prevalencia (53,1%) (2-4). Estas son cifras alarmantes debido a que el área rural concentra a la población con mayor pobreza y vulnerabilidad de nuestro país(1), situación que se ve empeorada con los efectos negativos que tienen la anemia y desnutrición crónica sobre el desarrollo cognitivo y la productividad de la población, lo que representa un escollo importante para lograr el desarrollo y superar la pobreza (5-8). En este contexto, la integración de la comunidad y la familia al desarrollo integral del niño en cuanto a metodologías podría tener un impacto importante en los indicadores de salud y desarrollo(9).

World Vision Perú (WVP) ha venido desarrollando intervenciones a favor de la infancia desde 2007, lo que ha permitido desarrollar una intervención conocida como Estrategia para el Crecimiento y Desarrollo Integral (ECDI), cuyo objetivo es mejorar el estado nutricional y desarrollo temprano de los niños menores de tres años de edad. El objetivo del presente estudio fue demostrar la efectividad de la ECDI para reducir la anemia y la desnutrición, y mejorar el desarrollo psicomotor y el cuidado y protección infantil en una zona rural del Perú.

## MATERIALES Y MÉTODOS

Se realizó un estudio cuasiexperimental en tres distritos rurales de la región Ayacucho de características geográficas, demográficas, sociales y de bienestar infantil similares, incluida la presencia de programas sociales entre los que destacan Juntos (transferencia condicionada de recursos) y Cuna Más (cuidado de niños menores de tres años y acompañamiento familiar en puericultura). El grupo de intervención estuvo conformado por las comunidades de los distritos de Acocro y Tambillo, donde funcionaba desde hacía uno a dos años un servicio integral infantil llamado, por WVP, Centro de Desarrollo Integral (CDI). El grupo de comparación estuvo conformado por las comunidades del distrito de Chiara.

El tamaño de muestra fue de 125 niños para cada grupo de estudio. Para su cálculo se consideró una diferencia igual o superior al 10% de la reducción del porcentaje de anemia entre ambos grupos, un nivel de confianza de 95%, un poder estadístico de 80% y 10% de pérdida de seguimiento.

Se incluyeron en el estudio a todos los niños que residían en las comunidades de estudio y que cumplieron con los siguientes criterios al inicio del estudio: menores de 36 meses de edad, cuyos padres residían por lo menos desde hacía un año en las comunidades seleccionadas, que vivían con su madre y cuyos padres brindaron consentimiento informado escrito. Se excluyeron los niños con antecedentes de enfermedad crónica y niños con padres con antecedente de enfermedad psiquiátrica o drogodependencia.

Los niños seleccionados fueron evaluados tres veces. La línea basal se realizó a fines de mayo de 2013; la medición intermedia, a fines de noviembre de 2013; y la medición final, a fines de mayo de 2014.

### Modelo innovador ECDI

El modelo basado en la ECDI incluyó el abordaje a nivel familiar y comunitario. A nivel familiar, se buscó fortalecer las capacidades de las familias a través de talleres en salud preventiva, nutrición, desarrollo temprano y protección infantil. Estas actividades fueron reforzadas con visitas domiciliarias y consejería oportuna y dirigida. A nivel comunitario, la intervención estuvo dirigida a la implementación de una vigilancia comunitaria del crecimiento y desarrollo infantiles, a cargo de las familias y con el acompañamiento de promotoras comunitarias o “madres guía”, quienes además realizaron el monitoreo del consumo de multimicronutrientes por los niños. Asimismo, la estrategia incluyó el acondicionamiento de los CDI con material lúdico, para fomentar el desarrollo integral del niño usando el enfoque del desarrollo autónomo de Pikler (10-12).

Se consideró que un niño tenía anemia cuando la concentración de hemoglobina (Hb) en su sangre fue menor de 11 g/dL, una vez que el resultado fue ajustado para la altitud de residencia del niño. Para ello se usó la fórmula: Hb ajustada = Hb observada – 0,032 × (alt) + 0,022 × alt2, donde (alt) es: (altura en metros sobre el nivel del mar/1 000) × 3,3(13). Se consideró que un niño tenía anemia grave cuando la Hb fue menor de 7 g/dL. La medición de la Hb se realizó a partir de los seis meses de edad, a través de la obtención de una muestra de sangre capilar mediante punción en la parte superior externa del dedo anular, tomando como muestra la tercera gota. Para el procesamiento de la muestra de sangre se utilizó el analizador HemoCue®.

Las mediciones antropométricas fueron realizadas por dos trabajadores de salud acreditados para realizar estas mediciones. Se utilizaron tallímetros de madera y balanzas debidamente certificados por el Centro Nacional de Alimentación y Nutrición (CENAN). Para el cálculo y evaluación de los indicadores antropométricos se usaron las tablas de la Organización Mundial de la Salud(14).

Para la evaluación psicomotriz se utilizó la pauta breve o test abreviado (TA) del Ministerio de Salud (MINSA), que evalúa cuatro áreas: lenguaje, motricidad, coordinación y social(15). Fue aplicado por el mismo personal profesional que realizó las mediciones antropométricas.

Además se aplicó una encuesta familiar, dirigida a las madres o cuidadoras del niño, que recogió datos sobre composición familiar, estado de salud del niño, alimentación del niño, incluido el listado de alimentos recibidos el día anterior y la frecuencia semanal de consumo de alimentos ricos en hierro (carnes, vísceras, menestras) y de facilitadores (cítricos, papaya, tomate) o inhibidores (infusiones, café, gaseosas) de absorción de hierro; actividad económica y protección (maltrato infantil) y cuidado del niño.

Para la sistematización y análisis de los datos se utilizó los programas Excel 2007 (Microsoft, WA, EE.UU.), SPSS v.20 (IBM Corp., NY, EE.UU.) y Epidat v.3.1 (Dirección Xeral de Saúde Pública, Consellería de Sanidade-Xunta Galicia, España; Organización Panamericana de la Salud, Washington DC, EE.UU.). Los datos de antropometría fueron procesados en el software Anthro de la OMS.

Para el análisis descriptivo de variables se calcularon porcentajes, prevalencias, medias y desviaciones estándares, según correspondiera. La asociación de variables cualitativas fue medida mediante las pruebas de independencia y de tendencia chi cuadrado de Pearson, así como la prueba de diferencia de proporciones para muestras independientes. Para las variables cuantitativas se aplicaron pruebas de comparación de grupos independientes.

Para evaluar los factores asociados con los efectos de interés, se utilizaron modelos de ecuaciones de estimación generalizadas (GEE), asumiendo una distribución binomial para la variable efecto, función de enlace logit y una estructura de autocovarianzas de tipo AR(1). Para todos los análisis estadísticos se utilizó un nivel de significación de 0,05.

Antes de realizar la recolección de los datos, se realizaron visitas de campo y coordinaciones con los gobiernos locales, establecimientos de salud y autoridades comunales de la zona, de quienes se obtuvo la autorización escrita para realizar el estudio. Además, se obtuvo el consentimiento informado de la madre, padre o tutor del niño. Los nombres de los participantes solo fueron accesibles a los investigadores del estudio y fueron guardados de manera segura (bajo llave las fichas y con clave la base de datos) en el local de WVP. Los participantes con anemia, desnutrición o retraso en el desarrollo psicomotor, fueron derivados al personal de salud del nivel local para tratamiento y seguimiento según los protocolos del MINSA. El estudio fue aprobado por el Comité de Ética del Instituto de Medicina Tropical de la Universidad Nacional Mayor de San Marcos.

## RESULTADOS

En el análisis de búsqueda de factores asociados se incluyeron 283 niños (126 del grupo de intervención y 157 del grupo de comparación) que tuvieron dos mediciones, incluida la final. Para el ajuste del modelo de regresión GEE múltiple se incluyeron los 205 niños que tuvieron las tres mediciones, 97 fueron del grupo de intervención y 108 del grupo control.

Las características sociodemográficas de las madres y niños de los dos grupos de estudio fueron semejantes al inicio y durante todo el estudio (cuadro 1).

La edad de los niños incluidos en la línea basal tuvo un media (desviación estándar) de 14,2 (8,6) meses; en la medición intermedia fue de 17,8 (10,0) meses; en la medición final fue de 24,1 (10,0) meses; no hubo diferencias entre grupos en ninguna de las mediciones. Para el grupo de 205 niños con tres mediciones, las medias para las mediciones basal, intermedia y final fueron 14,6 (8,4) meses, 20,5 (8,4) meses y 26,5 (8,4) meses respectivamente, sin diferencias entre los grupos de estudio para todas las mediciones.

La distribución por sexo fue semejante entre las mediciones realizadas: el porcentaje de niñas fue de 49,3% en la línea basal, 48,9% en la medición intermedia y 48,8% en la final, sin que hubieran diferencias entre grupos de estudio en cada medición. El porcentaje de niñas en el grupo con las tres mediciones fue de 49,8%.

### Cambios en la anemia y en el desarrollo temprano

La prevalencia de anemia fue muy alta en todas las mediciones, 85,6% en la medición basal, 70,6% en la intermedia y 59,0% en la final, pero con una tendencia significativa a la disminución (P < 0,001). En los niños con tres mediciones, tuvo el mismo comportamiento: 85,7%, 71,7% y 59,4% respectivamente (cuadro 2).

**FIGURA 1.**
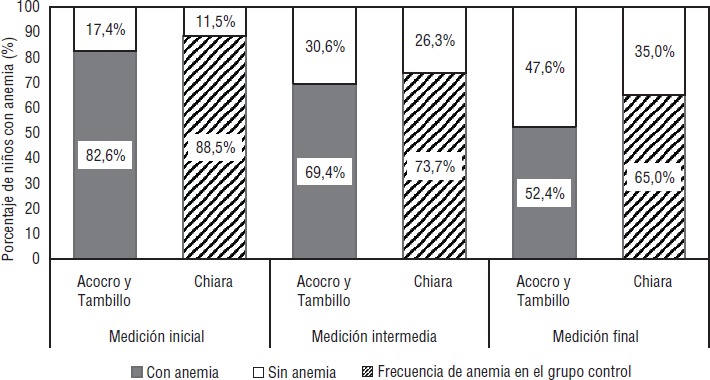
Porcentaje de anemia por grupo de estudio y medición. Años 2013–2014

En las comunidades de intervención se observó este mismo comportamiento, con una tendencia significativa a la disminución de la anemia (P < 0,001) y con una reducción de 36,5% (IC95%: 23,1%-47,7%) entre la medición basal y la final. En las comunidades control también hubo una tendencia significativa a la disminución (P < 0,001) y una reducción de 26,4% (IC95%: 16,0%-35,9%) del porcentaje de anemia basal-final. Al comparar la frecuencia de anemia entre los grupos de intervención y control, solo se encontró diferencias significativas en la medición final: 52,4% frente a 65,0%, *P* = 0,032 (figura 1).

**CUADRO 1.** Características sociodemográficas basales de los niños estudiados y sus padres. Acocro, Tambillo y Chiara, mayo 2013

Características	Intervención			Comparación		
	%	n	IC (95%)	%	n	IC (95%)
Sexo (femenino)	51,9	54	41,8 - 62,0	47,5	58	38,3 - 56,8
Grupo etario					
0 a 5 meses	17,3	18	9,6 - 25,1	21,3	26	13,6 - 29,0
6 a 11 meses	22,1	23	13,7 - 30,6	20,5	25	12,9 - 28,1
12 a 23 meses	42,3	44	32,3 - 52,3	38,5	47	29,5 - 47,6
24 a 35 meses	18,3	19	10,4 - 26,2	19,7	24	12,2 - 27,1
DNI del niño	94,1	96	89,1 - 99,2	93,4	114	88,6 - 98,2
Aseguramiento (SIS)	89,8	88	83,3 - 96,3	87,6	106	81,3 - 93,9
Características de la vivienda					
Vivienda independiente	98,0	98	93,0 - 99,8	95,9	116	90,6 - 98,6
Paredes de adobe	97,1	101	91,8 - 99,4	95,0	115	90,8 - 99,3
Techo de tejas, calamina o eternit	96,2	100	90,4 - 98,9	96,7	117	91,7 - 99,1
Habitaciones para dormir ( $\bar{x}$ )	1,5		1,3 - 1,6	1,5		1,3- 1,6
Alumbrado eléctrico	78,8	82	70,5 - 87,2	62,8	76	53,8 - 71,8
Red de agua dentro de la vivienda	57,7	60	47,7 - 67,7	64,5	78	55,5 - 73,4
Desagüe: pozo ciego o séptico	73,1	76	64,1 - 82,1	79,3	96	71,7 - 87,0
Edad de la madre ( $\bar{x}$ )	27,9		26,5 - 29,4	28,1		26,7- 29,4
Edad del padre ( $\bar{x}$ )	30,5		28,8 - 32,1	30,7		29,2- 32,3
Estado civil de la madre (unida)	87,5	91	80,7 - 94,3	86,9	106	80,5 - 93,3
Religión (católica)	63,8	60	53,6 - 74,1	79,3	96	71,7 - 87,0
Nivel de instrucción de la madre					
Primaria incompleta o ninguno	40,4	42	30,5 - 50,3	36,9	45	27,9 - 45,9
Primaria completa	9,8	19	5,4 - 14,2	20,5	25	12,9 - 28,1
Secundaria incompleta	28,8	30	19,7 - 38,0	32,8	40	24,0 - 41,5
Secundaria completa	10,6	11	4,2 - 17,0	7,4	9	2,3 - 12,4
Superior	1,9	2	0,2 - 6,8	2,5	3	0,5 - 7,0
Idioma principal de la madre					
Español	19,8	18	11,0 - 28,5	9,3	10	3,4 - 15,3
Quechua	80,2	73	71,5 - 89,0	90,7	97	84,7 - 96,6
Ingreso familiar en USD ( $\bar{x}$ )	230	517	200 - 265	225	559	193 - 257

$\bar{x}$ , promedio; IC95%, intervalo de confianza de 95%; DNI, documento nacional de identidad; SIS, seguro integral de salud; USD, dólares estadounidenses.

***Fuente:*** elaboración propia.

**CUADRO 2.** Prevalencia de anemia por grupo de edad y medición. Años 2013–2014

Grupo etario	Medición basal^a^		Medición intermedia^b^		Medición final^c^		Diferencia final-basal	
	%	n/N	%	n/N	%	n/N	%	IC95%
6-11 meses	93.8	45/48	90.7	39/43	89.2	33/37	4.9	8,8 - 16,8
12-23 meses	85.6	77/90	69	60/87	62.1	64/103	27.4	13,7 - 38,9
24 meses y más^d^	76.7	33/43	62.6	57/91	49	70/143	36.3	19,3 - 49,6
Total	85.6	155/181	70.6	156/221	59	167/283	31.1	22,8 - 38,5

Chi cuadrado de tendencia: ^a^P < 0,021; ^b^P < 0,001; ^c^P < 0,001; ^d^P < 0001.

El modelo de regresión (cuadro 3) mostró que la intervención ECDI tuvo un efecto significativo en la reducción de la anemia, al igual que el consumo adecuado de potenciadores de la absorción de hierro, la edad y sexo de los niños. Estos resultados fueron independientes del consumo de alimentos ricos en hierro, del consumo de inhibidores de hierro, del consumo de suplementos de hierro en los últimos seis meses y de la participación en el programa Cuna Más. La efectividad de la intervención para reducir la anemia fue de 33,1% (IC95%: 1,0%-54,7%).

La prevalencia de anemia fue mayor en los niños de 6 a 9 meses de edad, con tendencia a disminuir con la edad y entre mediciones (cuadro 2). El modelo de regresión mostró una disminución significativa de 5,8% (IC95%: 3,8%-7,7%) de la frecuencia de anemia por cada mes de edad de incremento (cuadro 3).

La prevalencia de anemia también tuvo una tendencia significativa a la disminución entre mediciones tanto para el sexo masculino (*P* < 0,001) como para el sexo femenino (*P* < 0,001), con reducción del 28,1% (IC95%: 17,3%-37,6%) del porcentaje de anemia basal-final en varones y del 33,7% (IC95%: 20,6%-44,7%) en las niñas (cuadro 4). El análisis de regresión mostró un mayor riesgo de anemia para los varones en comparación con las niñas, con un incremento del riesgo de anemia de 37,5% (IC95%: 7,5%-57,7%).

En relación a los hábitos alimenticios, solo el consumo adecuado de potenciadores de la absorción de hierro (cinco veces a la semana o más) mostró efecto protector para anemia. El consumo adecuado mejoró a predominio del grupo de intervención: pasó de 39,5% en la línea basal a 93,8% en la final (50,0% y 92,9% en el grupo con tres mediciones), mientras que en el grupo de comparación pasó de 72,8% a 82,2%, respectivamente (72,0% a 82,2% en niños con tres mediciones). El análisis de regresión mostró que se redujo el riesgo de anemia en 46,5% (20,7%-63,9%). No estuvieron asociados con la anemia, el consumo adecuado de inhibidores de hierro (dos veces a la semana o menos), el consumo adecuado de alimentos ricos en hierro (cinco veces a la semana o más) ni el consumo de sulfato ferroso o de multimicronutrientes en los últimos seis meses.

***Nota:*** Grupo intervención: Acocro y Tambillo; grupo control: Chiara.

***Fuente:*** elaboración propia.

**CUADRO 3.** Modelo de regresión de anemia ajustado para variables seleccionadas

Variables	OR	IC95%	Valor de P
Grupo (intervención/comparación)	0,669	0,453 - 0,990	0,044
Sexo (masculino/femenino)	1,599	1,081 - 2,364	0,019
Edad (meses)	0,942	0,922 - 0,962	0,000
Consumo alimentos ricos en hierro (adecuado/inadecuado)	1,094	0,571 - 2,095	0,787
Consumo potenciadores del hierro (adecuado/inadecuado)	0,535	0,361 - 0,793	0,002
Consumo inhibidores del hierro (adecuado/inadecuado)	0,815	0,546 - 1,218	0,319
Recibieron suplemento de hierro últimos seis meses (sí/no)	0,849	0,529 - 1,365	0,500
Cuna Más (sí/no)	1,155	0,783 - 1,703	0,468

OR, razón de momios (ajustado); IC95%, intervalo de confianza de 95%.

***Fuente:*** elaboración propia.

**CUADRO 4.** Prevalencia de anemia por sexo y medición. Años 2013–2014

Sexo^a^	Medición basal		Medición intermedia		Medición final	
	%	n/N	%	n/N	%	n/N
Masculino-intervención	82,5	33/40	80,7b	46/57	56,9	37/65
Femenino-intervención	82,6	38/46	56,9b	29/51	47,5	29/61
Masculino-control	94,3	50/53	73,3	44/60	70,0	56/80
Femenino-control	81,4	35/43	74,0	43/58	59,7	46/77
Masculino-total	89,2	83/93	76,9	90/117	64,1	93/145
Femenino-total	82,0	73/89	66,1	72/109	54,3	75/138

Chi cuadrado de tendencia: ^a^P < 0,010; ^b^P < 0,010.

***Fuente:*** elaboración propia.

### Cambios en los indicadores antropométricos

La frecuencia de desnutrición crónica (talla/edad) se mantuvo alrededor de 34% en todas las mediciones y en ambos grupos; de la misma manera, la desnutrición crónica grave se mantuvo en aproximadamente 7% de los niños.

La desnutrición aguda (peso/talla) estuvo presente en un niño (0,4%) en la línea basal, en ningún niño en la medición intermedia y en dos niños (0,7%) en la medición final, todos del grupo control. Asimismo, las frecuencias de sobrepeso y obesidad fueron de 2,2% y 1,3% en la línea basal, 1,1% y 0,0% tanto en la medición intermedia como en la final; no hubo diferencias entre grupos de estudio.

La desnutrición global (peso/edad) estuvo presente en aproximadamente 9% de los niños, sin diferencias entre grupos ni mediciones. El peso bajo grave se observó en aproximadamente 1% de los niños.

### Cambios en el desarrollo psicomotor

El porcentaje de niños con patrón de normalidad, según la prueba de desarrollo psicomotor, varió en el grupo intervención de 66,3% en la línea basal a 94,9% en la medición intermedia y 74,6% en la final; en el grupo control varió de 68,0% a 97,9% y 86,6%, respectivamente. No se encontraron diferencias significativas entre los grupos de estudio.

### Cambios observados en la protección y el cuidado del niño

La frecuencia de maltrato infantil tuvo un ligero incremento entre la medición basal y la final (29,7% frente a 32,9%). No obstante, la frecuencia de maltrato físico presentó una disminución al comparar la medición basal y final (14,2% frente a 8,6%, *P* = 0,064), mientras que el maltrato solo verbal se incrementó (15,5% frente a 24,3%, *P* = 0,020). No hubo diferencias entre los grupos de intervención y control. Asimismo, el maltrato fue más frecuente en los varones, aunque esta diferencia no fue significativa.

En cuanto al juego con los niños, fueron los hermanos mayores los que dedicaron mayor tiempo, seguidos de la madre. El porcentaje llegó a 50,0% en el grupo intervención y 39,5% en el de comparación, aunque sin diferencia entre grupos. En la medición final en el grupo de intervención, el porcentaje de ambos padres e hijos mayores que se involucraron en el juego con el niño fue de 67,5%, mientras que en el grupo control fue de 52,6%, diferencia que fue significativa (*P* = 0,024).

### Cambios en acceso al programa Cuna Más

Alrededor del 50% de los niños de ambos grupos eran beneficiarios del programa social Cuna Más, sin diferencias en el acceso entre mediciones.

## DISCUSIÓN

La intervención ECDI mostró un efecto significativo sobre la anemia al lograr una mayor reducción en el grupo de intervención en comparación con el grupo control. Sin embargo, la anemia se mantuvo durante todo el estudio por encima de 40,0%, lo que, de acuerdo con la OMS, representa un problema de salud pública muy grave (16-18). Esta prevalencia es mayor que la estimada para el nivel nacional para el mismo grupo de edad en 2013 (46,4%) y 2014 (46,8%), mayor que la encontrada en 2013 en el ámbito del programa Juntos (52,4%) y que la encontrada para la Región Ayacucho (45,8%) (19, 20). Esta situación es más grave en los niños entre los 6 y 11 meses, pues casi todos eran anémicos, aun cuando 92% de los niños en el grupo de intervención y 94% en el grupo control recibieron lactancia materna exclusiva hasta los cinco a seis meses de edad. Consideramos que esto es indicativo de inadecuadas reservas de hierro al momento del nacimiento, además de una deficiente ingesta de hierro durante la ablactancia. Es probable que el problema de la anemia en estos niños comience con la deficiencia de hierro o anemia en sus madres aún antes del comienzo de la gestación y con la inadecuada ingesta de hierro en el embarazo (4, 21-23).

Además, se pudo apreciar que el riesgo de anemia fue mayor en niños de sexo masculino, hecho que ha sido previamente observado en otros estudios y que se podría deber a una mayor necesidad de hierro para su crecimiento (4, 24, 25).

Si bien durante el estudio la suplementación con hierro se realizó por seis meses continuos, de acuerdo a las disposiciones del MINSA, al final de esta investigación la frecuencia de anemia continuó siendo muy alta en ambos grupos de estudio. Aunque las guías actuales de suplementación con hierro del MINSA indican que la suplementación preventiva debe realizarse por el lapso de 12 meses continuos en los niños de 6 a 36 meses de edad(26), consideramos que, para el caso de comunidades como las estudiadas, la suplementación debería mantenerse de manera continua durante los primeros 24 meses, como lo recomendaba antes la OMS(27), inclusive hasta los 36 meses de vida y debería comenzar aún antes de la ablactancia, a partir del mes de edad, tal como el MINSA lo recomienda para grupos de riesgo especiales como los niños de bajo peso al nacer y los prematuros(26). Por otra parte, el adecuado control del embarazo y la suplementación con hierro de las gestantes debería ser una prioridad en estos ámbitos, así como la implementación de otras medidas tales como el pinzamiento tardío del cordón umbilical durante el parto, la desparasitación de los niños, la consejería en temas nutricionales y el seguimiento y vigilancia comunitaria (4, 22, 28, 29).

Estimamos que la intervención ECDI fue exitosa para el control de la anemia por los talleres educativos realizados, orientados a la mejora de los hábitos nutricionales y el cuidado del niño, y por la implementación de un sistema de seguimiento y vigilancia comunitaria por madres guía que permitió, por ejemplo, usar un calendario tarjetero para objetivar el consumo de los sobres de multimicronutrientes.

Aproximadamente la tercera parte de los niños estudiados padecían desnutrición crónica, sin diferencias entre los grupos de estudio. Al analizar por grupo de edad, en la medición final, el grupo de 12 a 23 meses tuvo la mayor frecuencia (33,0%), lo que se asemeja a lo encontrado en otros estudios, donde la mayor prevalencia se presenta en el segundo año de vida(30). Intervenciones educativas nutricionales de mayor duración han logrado reducir la desnutrición crónica en el Perú y otros países (30-32), lo que podría explicar la ausencia de impacto de nuestra intervención. Nuestros resultados sugieren que hay un inadecuado manejo de la lactancia materna y de la ablactancia, así como inapropiados hábitos alimenticios que no cubren los requerimientos nutricionales de los menores de 36 meses de edad.

En cuanto al desarrollo psicomotor, no se encontraron diferencias entre grupos, aunque el porcentaje de niños con una evaluación normal se incrementó entre mediciones, especialmente en la medición intermedia, lo que podría haber sido resultado de un sesgo de medición por el instrumento utilizado, el test abreviado (TA)(15). Este es un instrumento basado en un enfoque conductista, que al ser invasivo a la espontaneidad del niño, puede generar en él una limitada respuesta frente a los estímulos que implica este tipo de evaluación del desarrollo. Por ello, hoy en día se prefiere el uso de otros métodos que privilegian la observación en un entorno conocido para el niño (10-12). La ausencia de efecto de la ECDI podría ser resultado de que el modelo de intervención basado en la teoría pikleriana requiere de un mayor tiempo para su adecuada implementación.

En relación al cuidado y protección del niño, el maltrato verbal mostró un incremento en los niños mayores en ambos grupos. Por otro lado, el maltrato físico tuvo tendencia a la disminución, aunque menos marcada en los varones. Otros estudios han encontrado también que los varones tienen mayor probabilidad de sufrir violencia física que las niñas (33-35).

El estudio tiene ciertas limitaciones. Hubo un grupo de 78 (27,5%) niños que no tuvieron las tres mediciones, principalmente por un tema de migración estacional de sus padres, relacionada a sus actividades productivas. No obstante, las características demográficas y de morbilidad de estos niños no difirieron de los niños que tuvieron tres mediciones, por lo que consideramos que su ausencia no compromete los resultados del estudio. Asimismo, el diseño no aleatorizado, sin técnicas de enmascaramiento podría haber introducido confusores difíciles de controlar. Sin embargo, creemos que la robustez de la metodología de selección de las comunidades, así como de medición de las variables de interés y de análisis estadístico le da solidez a los resultados.

Consideramos que la ECDI fue efectiva para mejorar la nutrición de los menores de 36 meses de edad a través de la reducción de la anemia y el incremento del consumo de potenciadores de la absorción de hierro. Las intervenciones que incluyen componentes educativos y de seguimiento comunitarios, podrían ser de gran ayuda para combatir la anemia en los niños menores de 36 meses de edad en comunidades rurales y, potencialmente, mejorar también otros indicadores nutricionales, del desarrollo psicomotor y cuidado y protección de los niños.

### Agradecimientos.

A los alcaldes distritales de Acocro, Tambillo y Chiara, a los alcaldes de los centros poblados, presidentes y demás autoridades de las comunidades estudiadas. A los jefes y personal de los establecimientos de salud de los distritos de Tambillo, Chiara y Acocro, quienes brindaron las facilidades de sus espacios para realizar la evaluación antropométrica y dosaje de hemoglobina a las niñas y niños del estudio: Javier Salazar (Guayacondo), Sonia Yauri (Sachabamba), Ulises Quispe (Manallasacc), Efraín Zegarra (Quishuarcancha), Angelino Cayllahua (Chiara) y Eduardo Aranguren (Acocro). A los funcionarios de la Municipalidad distrital de Chiara, Nilo Huaytalla y Mario Quispe, por su apoyo en las convocatorias en las comunidades de Manallasacc, Sachabamba, Quishuarcancha, Liriopata, Qhishuar, Intihuasi y Chiara, y el apoyo logístico durante la recolección de datos. A las facilitadoras comunitarias del Servicio de Acompañamiento de Familias del Programa Nacional Cuna Más y las presidentas del Programa Nacional Juntos, por su apoyo en la convocatoria a las madres de familia de su jurisdicción. A Sonia Shishido (UNMSM), Ramón J. Soto (LACRO) y Annette Ghee (Centro Global de World Vision International), por su asesoramiento y acompañamiento en diversas etapas de la investigación.

### Financiamiento.

El trabajo fue financiado por la Fundación Optimus UBS de Suiza y la oficina de World Vision en Suiza. Los patrocinadores no participaron de ninguna manera en el diseño del estudio, la recolección y análisis de los datos, la decisión de publicar este trabajo ni en la preparación del manuscrito.
